# Venomics and Cellular Toxicity of Thai Pit Vipers (*Trimeresurus macrops* and *T. hageni*)

**DOI:** 10.3390/toxins12010054

**Published:** 2020-01-16

**Authors:** Supeecha Kumkate, Lawan Chanhome, Tipparat Thiangtrongjit, Jureeporn Noiphrom, Panithi Laoungboa, Orawan Khow, Taksa Vasaruchapong, Siravit Sitprija, Narongsak Chaiyabutr, Onrapak Reamtong

**Affiliations:** 1 Department of Biology, Faculty of Science, Mahidol University, Ratchathewi, Bangkok 10400, Thailand; supeecha.kum@mahidol.edu (S.K.); siravit.sit@mahidol.ac.th (S.S.); 2 Snake Farm, Queen Saovabha Memorial Institute, The Thai Red Cross Society, Pathumwan, Bangkok 10330, Thailand; lchanhome@yahoo.com (L.C.); panithivet@gmail.com (P.L.); taksa.v@gmail.com (T.V.); 3 Department of Molecular Tropical Medicine and Genetics, Faculty of Tropical Medicine, Mahidol University, Ratchathewi, Bangkok 10400, Thailand; tipparat.thi@mahidol.ac.th; 4 Department of Research and Development, Queen Saovabha Memorial Institute, The Thai Red Cross Society, Pathumwan, Bangkok 10330, Thailand; tu-juree-n@hotmail.com (J.N.); okhow_2000@yahoo.com (O.K.)

**Keywords:** *Trimeresurus macrops*, *Trimeresurus hageni*, pit vipers, snake venom proteomics, cytotoxicity, U937 monocytes, fibroblasts

## Abstract

The two venomous pit vipers, *Trimeresurus macrops* and *T. hageni*, are distributed throughout Thailand, although their abundance varies among different areas. No species-specific antivenom is available for their bite victims, and the only recorded treatment method is a horse antivenom raised against *T. albolabris* crude venom. To facilitate assessment of the cross-reactivity of heterologous antivenoms, protein profiles of *T. macrops* and *T. hageni* venoms were explored using mass-spectrometry-based proteomics. The results show that 185 and 216 proteins were identified from *T. macrops* and *T. hageni* venoms, respectively. Two major protein components in *T. macrops* and *T. hageni* venoms were snake venom serine protease and metalloproteinase. The toxicity of the venoms on human monocytes and skin fibroblasts was analyzed, and both showed a greater cytotoxic effect on fibroblasts than monocytic cells, with toxicity occurring in a dose-dependent rather than a time-dependent manner. Exploring the protein composition of snake venom leads to a better understanding of the envenoming of prey. Moreover, knowledge of pit viper venomics facilitates the selection of the optimum heterologous antivenoms for treating bite victims.

## 1. Introduction

Venomous pit vipers are snakes of the Crotalinae subfamily, characterized by two movable fangs and heat-sensing pit organs located bilaterally between the eye and nostril. *Trimeresurus* is a prominent pit viper genus and comprises the greatest number of known species [1]. *Trimeresurus* snakes are endemic to Asia; they are widely distributed, ranging from deserts to rainforests and in terrestrial, arboreal, and aquatic habitats. *Trimeresurus* bite victims have been reported in several geographic regions, including Lao PDR [2], Hong Kong [3], Taiwan [4], Thailand [5], China [6], Sri Lanka [7], and Japan [8]. In addition, green pit vipers were responsible for 58% of snakebites reported in Vietnam in 2017 [9]. *Trimeresurus* venom varies in toxicity between species; prolonged clotting time is a significant symptom observed in humans [10], and tissue damage and hematotoxicity in bite victims have also been reported [11,12]. Since there is no species-specific antivenom available for *Trimeresurus*, except *T. albolabris*, the only treatment available for bite cases has been a hetero-specific antivenom [13]. Antivenoms raised in horses are the most common therapeutic agents for snakebite treatment; however, they can cause several side effects, such as anaphylactic shock and serum sickness [14]. Moreover, preparation of antivenom from horse blood is laborious and time-consuming with a low production yield [15]. According to proteomics studies, each specific venom contains a unique variety of toxins [16]. To date, *T. insularis* (Indonesian), *T. borneensis* (Borneo), *T. stejnegeri* (Taiwan), *T. puniceus* (Java), *T. purpureomaculatus* (Thailand), *T. gramineus* (India), *T. nebularis* (Malaysia), and *T. alborabris* (Thailand) have been investigated for their venom constituents [17,18,19]. However, there is no reported information on the venomic protein profile of the Southeast Asia endemic species *T. macrops* and *T. hageni*.

**Figure 1:**
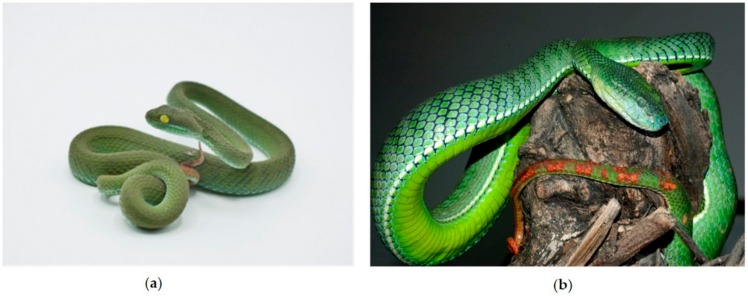
Two prominent species of *Trimeresurus* snakes in Thailand. Adult large-eyed pit viper (*T. macrops*) with its noticeably large eyes (**a**) and the Hagen’s pit viper (*T. hageni*) perching on a tree branch (**b**).

The large-eyed pit viper *T. macrops* can be distinguished from other green pit vipers by its relatively large eyes (Figure 1a). *T. macrops* bites frequently cause severe tissue damage in humans with the symptoms ranging from local swelling to severe systemic bleeding [20]. Its venom has a long half-life and can be retained within the human body for more than 14 days [21]. *T. hageni*, known as Hagen’s pit viper is also endemic to Southeast Asia (Figure 1b); however, there are only a few reports of the major symptoms of *T. hageni* venom. In addition, no *T. macrops* or *T. hageni* species-specific antivenoms are available. The antivenom raised from *T. albolabris* is currently used for neutralizing *T. macrops* and *T. hageni* venoms. In our study, a comparative proteomics approach was applied to the study of *T. macrops* and *T. hageni* venom protein composition. Moreover, we assessed the cytotoxicity of *T. macrops* and *T. hageni* venoms on monocytic cells (U937 cells) and skin fibroblasts (CRL-1474 cells) to clarify the effects on cellular physiology. The findings facilitate the analysis of cross-reactivity between these snake toxins and the available antivenoms. The identified toxins may be used for the development of inhibitory or neutralizing agents using molecular techniques to improve snakebite treatment. In addition, some proteins with beneficial activities could be further developed as novel pharmacological agents for human disease.

## 2. Results

### 2.1. Proteomics Analysis of T. macrops and T. hageni Venom

**Figure 2:**
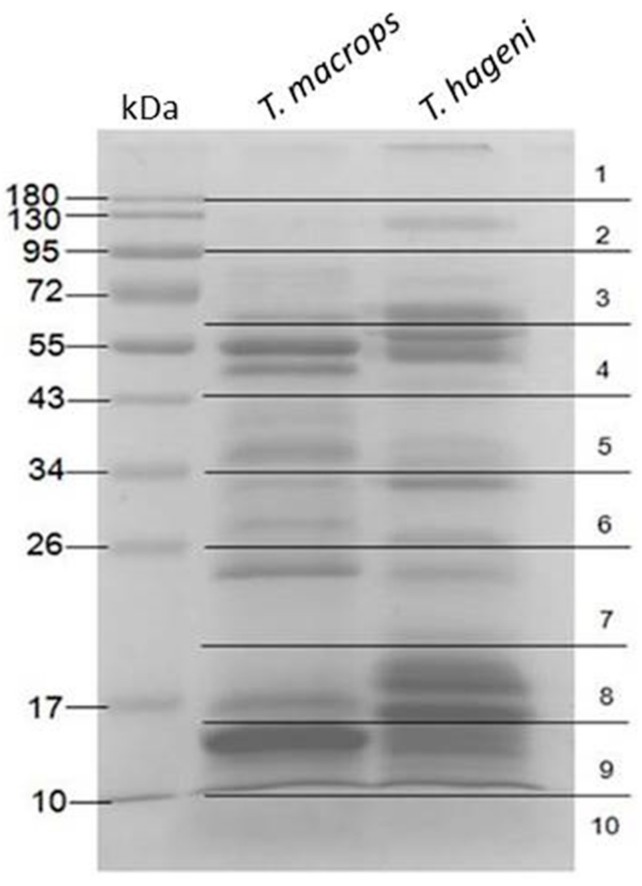
Coomassie blue-stained 12% sodium dodecyl sulfate polyacrylamide gel electrophoresis of *T. macrops* and *T. hageni* venoms (30 µg) under reducing conditions.

After preparing venom from *T. macrops* and *T. hageni*, the proteins were separated on 12% sodium dodecyl sulfate polyacrylamide gel electrophoresis (SDS-PAGE) (Figure 2). Bands at 15, 25, 35, 50, and 55 kDa were the most abundant in *T. macrops* venom. Whereas, bands at 15, 16, 20, 22, 32, 55, and 66 kDa were the most intense in *T. hageni* venom. Each gel lane was excised into 10 pieces. Peptides were extracted from the gel by in-gel digestion and further subjected to liquid chromatography–mass spectrometry (LC-MS/MS) analysis. After MASCOT searching against NCBI (Taxonomy: Chordata), the results revealed that the *T. macrops* and *T. hageni* venoms contained 185 and 216 proteins, respectively (Supplementary Table S1). The identified proteins were classified according to their gene ontology, including molecular function, biological process, and cellular component terms (Table 1).

**Figure 3:**
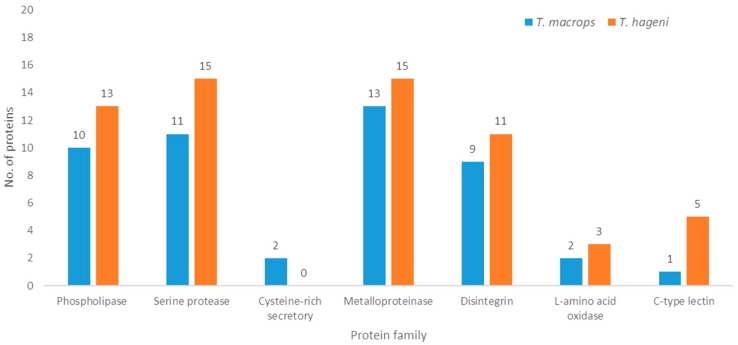
Proteome classification of *T. macrops* (blue) and *T. hageni* (orange) venoms; proteomes were classified according to common functions of snake venom.

In terms of molecular function, most *T. macrops* (75%) and *T. hageni* (44.4%) venom proteins were involved in catalytic activity. Structural molecule activity proteins were observed only in *T. macrops* venom and represented 12.5% of the proteins. Whereas, molecular function regulators comprised 22.2% of *T. hageni* venom proteins. In terms of biological processes, proteins involved in biological regulation and cellular processes were the largest classes present in *T. macrops* (36.4%) and *T. hageni* (26.7%) venoms, respectively. While immune system process, response to stimulus, and localization proteins were found only in *T. hageni* venom. In terms of cellular processes, organelle proteins were found in both *T. macrops* (37.5%) and *T. hageni* (50%) venoms. Whereas, membrane and protein-containing complex molecules were presented only in *T. macrops* venom. Phospholipase A2 (PLA2), snake venom serine protease (SVSP), cysteine-rich secretory, snake venom metalloproteinase (SVMP), disintegrin, L-amino acid oxidase, and C-type lectin were common to the two snake venoms. These protein families contribute to the phenotypic effects of venom on victims. Therefore, all identified proteins were also classified according to the common properties of snake venom, as shown in Figure 3. PLA2, SVSP, cysteine-rich secretory, SVMP, and disintegrin were more abundant in *T. macrops* venom. Whereas, L-amino acid oxidase and C-type lectin were more abundant in *T. hageni* venom. Due to protein semi-quantification, the exponentially modified protein abundance index (emPAI) was used to estimate the amount of proteins. The 20 most abundant proteins in *T. macrops* and *T. hageni* venoms are displayed in Table 2 and Table 3, respectively.

### 2.2. Cellular Toxicity of T. macrops and T. hageni Venoms on U937 Monocytic Cells and CRL-1474 Skin Fibroblasts

**Figure 4:**
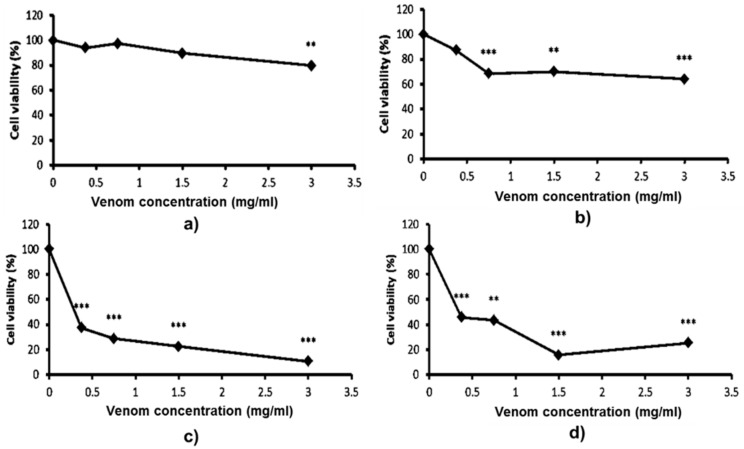
Cytotoxicity of *Trimeresurus macrops* and *T. hageni* venoms on human monocytic cells determined by 3-(4,5-dimethyl thiazol-2-yl)-2,5-diphenyl tetrazolium bromide (MTT) assay. U937 cells were treated with 0 to 3 mg/mL *T. macrops* venom for 48 (**a**) and 72 h (**b**) or with 0 to 3 mg/mL of *T. hageni* venom for 48 (**c**) and 72 h (**d**). Percentage U937 cell viability of venom-treated compared with non-venom treated cells (control). Data represent the mean ± standard error of mean (SEM) from three independent experiments, ** *p* < 0.01, *** *p* < 0.001.

At the physiological level, *Trimeresurus* venoms possess a hematotoxic effect; however, the influence of these venoms at the cellular level on immunopotent, blood-inhabited mononuclear cells remains unknown. The cytotoxic effects of *T. macrops* and *T. hageni* venoms on U937 monocytes was examined using the 3-(4,5-dimethyl thiazol-2-yl)-2,5-diphenyl tetrazolium bromide (MTT) assay (Figure 4). There was no remarkable cytotoxic of *T. macrops* venom on U937 cells (Figure 4a,b). Monocytic cells seemed to be more susceptible to *T. hageni* than *T. macrops* venoms, as a sharp decrease in viable cells could be detected at 24 h for 0.5 mg/mL (*p* < 0.001) (Figure 4c). The cytotoxicity increased as the concentration of *T. hageni* venom increased, indicating a concentration-dependent action. In addition, a similar toxicity pattern was found when U937 cells were exposed to this species’ venom for 72 h, and the LC_50_ values of *T. hageni were* 0.66 and 0.90 mg/mL at 48 and 72 h, respectively.

**Figure 5:**
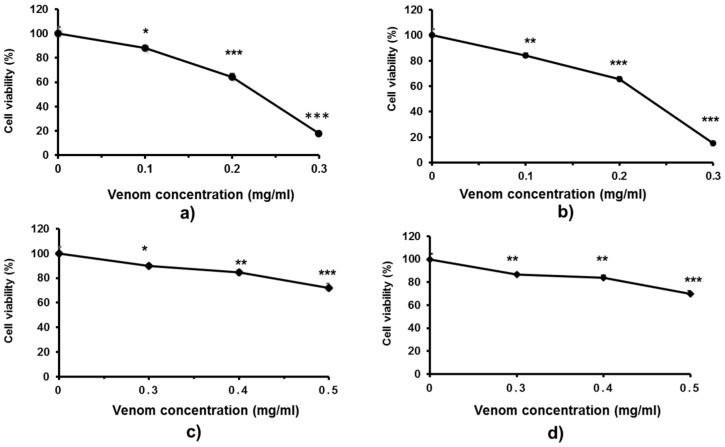
Cytotoxicity of Trimeresurus *macrops* and *T. hageni* venoms on human skin fibroblasts determined by 3-(4,5-dimethyl thiazol-2-yl)-2,5-diphenyl tetrazolium bromide assay. CRL-1474 skin fibroblasts were treated with 0 to 0.3 mg/mL *T. macrops* venom for 48 (**a**) and 72 h (**b**) or with 0 to 0.5 mg/mL *T. hageni* venom for 48 (**c**) and 72 h (**d**). Percentage CRL-1474 fibroblast cell viability of venom-treated compared with non-venom treated cells (control). Data represent the mean ± standard error of mean (SEM) from two independent experiments, * *p* < 0.05, ** *p* < 0.01 and *** *p* < 0.001.

Because skin is the first organ to encounter the venom after a snakebite and severe dermal tissue damage is frequently reported for *Trimeresurus* cases, the effects of these pit viper venoms on skin fibroblasts were investigated (Figure 5). In comparison with monocytic cells, fibroblasts were more sensitive to both *T. macrops* and *T. hageni* venoms. Fibroblast cell viability substantially reduced after exposure to 0.1 mg/mL of *T. macrops* at 48 h (*p* < 0.05), and the decline persisted as the concentration of venom increased (Figure 5a), and there was a comparable cytotoxicity at 72 h (Figure 5a,b). LC_50_ values of *T. macrops* were 0.23 mg/mL at both 48 and 72 h. Compared with *T. macrops*, *T. hageni* venom showed negligible toxicity to fibroblasts. (Figure 5c,d). Concerning the relatively high LC_50_ doses, it should be considered that crude venom have been used in these experiments and that there is possible low synergic biological effects among the enzymes and other proteins in the venom of both snakes.

## 3. Discussion

The protein patterns differed between *T. macrops* and *T. hageni* venoms, which was indicative of the non-identical protein composition of the two venoms. *T. macrops* and *T. hageni* venoms also demonstrated different protein patterns from *T. borneensis, T. gramineus*, *T. puniceus*, *T. purpureomaculatus*, *T. stejnegeri*, and *Protobothrops flavoviridis* [22]. All proteins of both venoms were classified according to their gene ontologies. In terms of molecular function, proteins involved in catalytic activity were the most abundant in both venoms, which corroborates the gene ontology analysis of platypus venom [23]. This protein class includes hemolytic and proteolytic proteins that play important roles during prey acquisition. The main functions of snake venom are to lubricate and immobilize prey. Several protein families have been found to be common in snake venoms, including phospholipase, serine protease, cysteine-rich secretory, metalloproteinase, disintegrin, L-amino acid oxidase, and c-type lectin. Our data show that in *T. hageni* venom, the abundance of proteins of the phospholipase, serine protease, metalloproteinase, disintegrin, L-amino acid oxidase, and c-type lectin families was higher than in *T. macrops* venom. However, no cysteine-rich secretory proteins were observed in *T. hageni* venom.

The two snake species produce biologically active substances in their venom capable of weakening prey to facilitate their capture. Phospholipase can hydrolyze glycerophospholipids to fatty acids and other lipophilic compounds [24]. This protein family from dangerous snake venoms has been reported to affect the peripheral neuromuscular system [25]. In addition, purified phospholipase A2 originating from various snake venoms have been shown to possess anticoagulant properties [26]. *Agkistrodon halys blomhoffii* [27] and *A. palas* [28] phospholipase A2 show antibacterial activity. The acidic phospholipase A2 5 (gi|13959432), acidic phospholipase A2 6 (gi|20177994), and basic phospholipase A2 2 (gi|13959429) were one of the twenty most abundant proteins in *T. macrops* crude venom. While, acidic phospholipase A2 2 (gi|3914268), acidic phospholipase A2 5 (gi|13959432), basic phospholipase A2 homolog Ts-R6 (gi|82201344), acidic phospholipase A2 1 (gi|129417), and D1E6b phospholipase A2 (gi|59727030) were among the 20 most abundant proteins in *T. hageni* crude venom. Although phospholipases were abundance proteins identified in both *T. macrops* and *T. hageni.* Their biological activities such as neuromuscular, anticoagulant, and antibacterial require further experiment for characterization.

Serine proteases have been reported to effect blood coagulation systems, e.g., the fibrinolytic system [29]. Some venom serine proteases are not susceptible to hirudin, heparin, or the most endogenous serine protease inhibitors. A few venom serine proteases can activate coagulation factor V, protein C, plasminogen, and platelets [30]. Snake venom serine protease 2B (gi|13959619) and snake venom serine protease 1 (gi|13959617) were found in abundant proportions in *T. macrops* and *T. hageni* crude venoms, respectively, and similarly to serine proteases of other snake venoms, they may disturb prey blood coagulation, resulting in severe bleeding. Venom metalloproteinases are also exceedingly toxic; these enzymes interfere with blood coagulation and hemostatic plug formation and destroy cell membranes and the extracellular matrix [31]. Snake venom metalloproteinase (gi|123894851) was abundant in *T. macrops* crude venom and may fulfil the same role as in other snake venoms.

Disintegrins are cysteine-rich peptides ranging from 45 to 84 amino acids in length and are non-enzymatic proteins found in the venom of numerous snake families. Their influence on platelet aggregation and integrin-dependent cell adhesion has been reported [32]. The proteolysis resistance of these proteins results in a sustained half-life in the blood of prey [33]. Disintegrin trigramin-gamma (gi|67462321) was predominantly found in *T. macrops* crude venom and plays a similar role as in other snake venoms. L-amino acid oxidase accelerates the oxidation of amino acids [34] and can cause plasma clotting disorders as well as inducing apoptosis in various cell lines [35]. L-amino acid oxidase was found in high concentrations in *T. macrops* and *T. hageni* and may contribute to the toxicity of the venoms. C-type lectin protein is a non-enzymatic, calcium dependent protein that binds sugar residues [36]. Many snake venom c-type lectin-like proteins target coagulation factors [37]. The Southeast Asian *T. albolabris* c-type lectin protein binds directly to the von Willebrand factor receptor, which has key functions in both the hemostatic and thrombotic pathways, leading to platelet agglutination [38]. In contrast, c-type lectin protein found in the Asian viper *Echis carinatus* inhibits platelet agglutination [39]. C-type lectin-like proteins were found in *T. macrops* and *T. hageni* venoms and may have similar functions in blood coagulation and platelet activation.

Cysteine-rich secretory protein is a single-chain bioactive polypeptide identified in several organisms, including snakes [40], reptiles [41], and mammals [42]. Snake venom cysteine-rich secretory protein can block smooth muscle contraction by blocking l-type calcium channels [43]. Moreover, this protein inhibits cyclic nucleotide-gated ion channels, which are significant in various sensory pathways, including vision and olfaction, as well as in other key cellular functions, such as hormone release and chemotaxis [44]. The cysteine-rich secretory protein family was identified in *T. hageni*, but not *T. macrops*, venom. The biological activity of these proteins may include effects on prey vision and olfaction.

Several studies have demonstrated the cytotoxicity of crude snake venom on human cell lines using in vitro assays, e.g., *Lachesis muta* venom on keratinocytes [45], and *Ophiophagus hannah* and *Echis carinatus* on pancreatic tumor cells [46]. Both *T. macrops* and *T. hageni* showed cytotoxicity towards human monocytes and skin fibroblasts. However, the latter exhibited a greater sensitivity to both *Trimeresurus* venoms, indicating a greater abundance of molecular targets for proteolytic enzymes. In addition, Foxo-3-mediated oxidative stress DNA damage leading to cellular senescence was recently reported in fibroblasts exposed to non-lethal doses of *Naja siamensis*, *Naja melanoleuca*, and *Dendroaspis viridis* venoms [47]. Such effects could also be stimulated by pit viper venoms.

There is an agreement between the monocyte toxicity and the proteomics results, as *T. hageni* contained more venomous biological substances, and the venom contained members of the cysteine-rich secretory protein family. Indeed, the discrepancy in fibrinogen pseudo-procoagulant clotting potency and Green Pit Viper antivenom neutralization efficacy between *T. macrops* and *T. hageni* was recently revealed [48], reflecting the fundamental evolutionary differences among the different *Trimeresurus* species. An in-depth study of other relevant physiologic effects would be beneficial in the continuing characterizations of the clinical effects of *T. macrops* and *T. hageni* venoms. The information would also facilitate the use of antivenom for treating bite victims. In addition, some venom components may be useful in the development of novel therapeutic agents for human diseases.

## 4. Materials and Methods

### 4.1. Venom Collection

The study has been reviewed and approved by the committee in accordance with Queen Saovabha Memorial Institute regulations and policies governing the care and use of laboratory animals (QSMI-ACUC-01-2018). The venoms of *T. macrops* and *T. hageni* were extracted from individual snakes and kept in a 1.5 mL microcentrifuge tubes. After being weighed, the fresh (liquid) venoms were immediately frozen at −20 °C and lyophilized. The dry (lyophilized) venoms were pooled (from at least three snakes) and stored at −20 °C for use in this study.

### 4.2. T. macrops and T. hageni Venom Protein Separation

Crude venoms of *T. macrops* and *T. hageni* were mixed with lysis buffer (containing 1% Triton X-100 (Merck, Germany), 1% sodium dodecyl sulfate (SDS) (Merck, Darmstadt, Germany), and 1% NaCl (Merck, Germany). The venoms were analyzed for their estimated protein concentration by Quick Start™ Bradford Protein Assay (Bio-Rad, Berkeley, CA, USA). A 30 µg sample of *T. macrops* and *T. hageni* venom solutions were separated by 12% SDS-PAGE (Bio-Rad, CA, USA) and stained by Coomassie R-250 solution (Bio-Rad, CA, USA). A whole lane of each venom was excised into 10 pieces and further subjected to in-gel digestion.

### 4.3. In-Gel Digestion

A 50% acetonitrile (ACN) in 50 mM ammonium bicarbonate solution was used for destaining the blue color from gel slides. Venom proteins were reduced by 4 mM dithiothreitol and incubated at 60 °C for 15 min. The reduced proteins were further alkylated by 250 mM iodoacetamine (Sigma-Aldrich, Saint Louis, MO, USA) and incubated at room temperature for 30 min in the dark. The gel pieces were dehydrated by removing all solution and adding 100% ACN (Thermo Scientific, Waltham, MA, USA). For tryptic digestion, trypsin (Sigma-Aldrich, USA, T6567) in 50 mM ammonium bicarbonate (Sigma-Aldrich, USA) was added to rehydrate the gels and incubated overnight at 37 °C. The peptide was extracted by adding 100% ACN and incubating for 15 min. The solution was transferred into a new microcentrifuge tube and dried using centrifugal concentrator (TOMY, Katsushika, Japan). The peptide mixtures were stored at −20 °C prior to mass spectrometric (MS) analysis.

### 4.4. Mass Spectrometric Analysis

Venom peptides were dissolved in 0.1% formic acid (Sigma-Aldrich, USA) and subjected to an Ultimate^®^ 3000 Nano-LC system analysis (Thermo Scientific, USA). The peptides were eluted and infused with a microTOF-Q II (Bruker, Bremen, Germany). The acquisition was operated by HyStar™ version 3.2 (Bruker, Germany). The data were processed and converted to mascot generics files (.mgf) using Compass DataAnalysis™ software version 3.4 (Bruker, Germany). The database search was performed using Mascot Daemon software (Matrix Science, Boston, MA, USA) against Chordata NCBI database with the following parameters: one missed cleavage site, variable modifications of carbamidomethyl (C) and oxidation (M), 0.8 Da for MS peptide tolerance, and 0.8 Da for MS/MS tolerance. The significance threshold was set at 95%. Three biological replications were performed for protein identification. The obtained proteins were classified according to their gene ontology using Blast2Go software.

### 4.5. Cell Culture

Human monocytic cells (U937 cell line from CLS cell line service) and normal human skin fibroblast cell line (CRL-1474 from ATCC) were cultured in RPMI 1640 (Invitrogen, Carlsbad, CA, USA) and D-MEM (Invitrogen), respectively. Media were supplemented with 10% fetal calf serum (Sigma-Aldrich, Saint Louis, MO, USA), 2 mM L-glutamine, penicillin (100 units/mL), and streptomycin (100 µg/mL). The cells were incubated in 5% CO_2_ at 37 °C and sub-cultured every 3 days. The morphology was monitored under an inverted microscope (CX1 Olympus, Tokyo, Japan).

### 4.6. Venom Cytotoxicity on Monocytic Cell (U937 Cell) and Skin Fibroblasts (CRL-1474)

U937 cells at a density of 2.5 × 10^5^ cells/mL and fibroblasts at 105 cells/mL were seeded into a well of 96-well plate and cultured for 24 h. The solution was discarded from each well. *T. macrops* and *T. hageni* crude venoms were added to U937 cells (at concentration ranging from 0 to 3 mg/mL), and fibroblasts (at concentration ranging from 0 to 0.5 mg/mL) and further incubated for 48 and 72 h. To examine cell viability, 10 µL of 5 mg/mL MTT was added to each well. The reaction was incubated in 5% CO_2_ at 37 °C for 4 h. The absorbance of formazan product was measured at 570 nm by an Infinite M200 PRO microplate reader (Tecan, Mannedorf, Switzerland). The percentage of cell viability was calculated relative to the untreated control cells.

### 4.7. Statistical Analysis

Quantitative data are presented as mean ± SEM. Statistical significance between groups was analyzed using standard t-tests or two-way ANOVA followed by the Bonferroni test. Significant *p*-values are indicated within the figure panels. Error bars indicate SEM.

## Figures and Tables

**Table 1:** Gene ontology classification of *Trimeresurus macrops* and *T. hageni* crude venom proteins.

Gene Ontology	% of Protein Components	
	*T* *. macrops*	*T* *. hageni*
Molecular Function		
binding (GO:0005488)	12.5	33.3
catalytic activity (GO:0003824)	75.0	44.4
structural molecule activity (GO:0005198)	12.5	-
molecular function regulator (GO:0098772)	-	22.2
Biological Process		
biological regulation (GO:0065007)	36.4	20.0
cellular process (GO:0009987)	27.3	26.7
metabolic process (GO:0008152)	9.1	6.7
rhythmic process (GO:0048511)	27.3	20.0
immune system process (GO:0002376)	-	6.7
response to stimulus (GO:0050896)	-	6.7
localization (GO:0051179)	-	13.3
Cellular component		
cell (GO:0005623)	25.0	33.3
extracellular region (GO:0005576)	12.5	16.7
membrane (GO:0016020)	12.5	-
organelle (GO:0043226)	37.5	50.0
protein-containing complex (GO:0032991)	12.5	-

**Table 2:** Top-20 unique proteins identified in *T. macrops* crude venom.

No.	Accession No.	Protein	Family	Score	M.W.	No. of Peptide	% Sequence Coverage	pI	emPAI
1	gi|299493	Thrombin-like proteinase	Protease	99	2737	2	54.2	4.31	4.69
2	gi|46395675	Cysteine-rich venom protein tripurin	Toxin activity	86	3971	2	33.3	9.19	2.76
3	gi|13959432	Acidic phospholipase A2 5	Toxin activity	442	13,870	4	27.9	4.72	2.74
4	gi|67462321	Disintegrin trigramin-gamma	Protease	111	7568	2	28.8	4.61	2.17
5	gi|697351484	Chain A, crystal structure of an acidic Pla2	Toxin activity	443	13,710	4	36.4	4.81	2.03
6	gi|60593434	Chain A, crystal structure of stecrisp	Toxin activity	238	25,112	4	18.1	5.53	1.12
7	gi|20177994	Acidic phospholipase A2 6	Toxin activity	146	15,712	3	21	4.72	0.79
10	gi|538259813	Galactose binding lectin, partial	CHO binding	79	17,654	3	23	5.72	0.69
11	gi|3552036	Pallase	Protease	151	26,162	3	19.4	5.89	0.62
12	gi|344268956	Phorbol-12-myristate-13-acetate-induced protein 1	Apoptotic process	36	6008	1	12.7	10.06	0.60
14	gi|13959429	Basic phospholipase A2 2	Toxin activity	130	7813	1	14.3	8.49	0.45
15	gi|82094948	Bradykinin-releasing enzyme KR-E-1	Protease	102	25,335	3	13.2	4.82	0.45
16	gi|123894851	Snake venom metalloproteinase	Protease	364	68,673	7	12.2	6.4	0.39
17	gi|13959619	Snake venom serine protease 2B	Protease	71	28,915	3	17.3	5.54	0.39
18	gi|538259847	5’-nucleotidase	Nucleotidase activity	203	57,055	7	15.1	8.27	0.32
19	gi|32469800	Thrombin-like enzyme contortrixobin	Protease	102	25,396	3	13.2	4.95	0.28
20	gi|143681919	Thrombin-like enzyme kangshuanmei	Protease	99	26,415	2	14.8	8.27	0.27

**Table 3:** Top-20 unique identified proteins from *T. hageni* crude venom.

No.	Accession No.	Protein	Family	Score	M.W.	No. of Peptides	%Sequence Coverage	pI	emPAI
1	gi|82130933	Snaclec coagulation factor IX-binding protein subunit A	Toxin activity	145	14,631	4	31.8	4.92	1.31
2	gi|67462321	Platelet aggregation activation inhibitor	Protease	162	7568	2	28.8	4.61	1.16
3	gi|60593434	Chain A, crystal structure of stecrisp	Toxin activity	174	25,112	5	21.7	5.53	1.12
4	gi|3914268	Acidic phospholipase A2 2	Toxin activity	155	13,784	4	38.5	4.95	0.94
5	gi|538259791	C-type lectin factor IX/X binding protein A subunit,	CHO binding	122	14,688	3	30	4.92	0.87
6	gi|32469800	Thrombin-like enzyme contortrixobin	Protease	450	25,396	6	18.4	4.95	0.85
7	gi|39655009	Chain A, crystal structure of a platelet agglutination factor isolated from the venom of Taiwan Habu	Protease	227	15,718	5	26.7	5.09	0.79
8	gi|126130	Galactose-specific lectin	CHO binding	55	16,281	4	31.1	5.54	0.76
9	gi|565235162	hypothetical protein L345_18387, partial	Unknown	36	5242	1	37.5	3.63	0.7
10	gi|13959617	Snake venom serine protease 1	Protease	150	27,923	6	22.5	5.66	0.57
11	gi|13959432	Acidic phospholipase A2 5	Toxin activity	65	13,870	2	11.5	4.72	0.55
12	gi|82201344	Basic phospholipase A2 homolog Ts-R6	Toxin activity	56	15,477	5	24.8	8.5	0.49
13	gi|129417	Acidic phospholipase A2 1	Toxin activity	62	15,524	2	10.1	6.54	0.48
14	gi|356581537	Beta-defensin-like protein 10	Chemokine receptor binding	14	7492	1	9	9.73	0.48
15	gi|59727030	D1E6b phospholipase A2	Toxin activity	85	15,959	2	10.1	4.79	0.47
16	gi|360797	Flavoxobin	Protease	158	25,725	5	13.6	5.2	0.44
17	gi|538259853	Phosphodiesterase	Magnesium binding	128	96,526	15	17	8.03	0.44
18	gi|676251620	Neurofibromin,	Phosphatid-ylcholine binding	30	7991	1	8.6	8.66	0.44
19	gi|538259847	5ʹ-nucleotidase,	Nucleotidase activity	177	57,055	10	19.5	8.27	0.4
20	gi|632991866	Transmembrane protein 184B-like	Unknown	52	9150	1	13.1	5.65	0.37

## Supplementary Materials

The following are available online at https://www.mdpi.com/2072-6651/12/1/54/s1, Supplementary Table S1. *T. macrops* and *T. hageni* venom proteins identified by proteomics (XLSX 558 KB).
